# Hydrogel spacer shrinkage during external-beam radiation therapy following low-dose-rate brachytherapy for high-risk prostate cancer: a case series  

**DOI:** 10.1186/s13256-021-02864-9

**Published:** 2021-05-26

**Authors:** Katsumaro Kubo, Masahiro Kenjo, Hideo Kawabata, Koichi Wadasaki, Mitsuru Kajiwara, Yoshiko Doi, Minoru Nakao, Hideharu Miura, Shuichi Ozawa, Yasushi Nagata

**Affiliations:** 1 Department of Radiation Oncology, Hiroshima University Hospital, 1-2-3 Kasumi Minami-ku Hiroshima-shi, Hiroshima, 734-8553 Japan; 2 Hiroshima High-Precision Radiotherapy Cancer Center, 2-2 Futabanosato Higashi-ku Hiroshima-shi, Hiroshima, 732-0057 Japan; 3 Department of Radiation Oncology, Hiroshima Prefectural Hospital, 1-5-54 Ujinakanda Minami-ku Hiroshima-shi, Hiroshima, 734-0004 Japan; 4 Department of Urology, Hiroshima Prefectural Hospital, 1-5-54 Ujinakanda Minami-ku Hiroshima-shi, Hiroshima, 734-0004 Japan

**Keywords:** Case report, Prostate cancer, Brachytherapy, External-beam radiation therapy, Hydrogel spacer

## Abstract

**Background:**

Few studies have assessed hydrogel spacer shrinkage during external-beam radiation therapy following brachytherapy for localized high-risk prostate cancer. This case presentation evaluated the changes in hydrogel spacer appearance by magnetic resonance imaging during external-beam radiation therapy after brachytherapy for prostate cancer and analyzed the effect of this shrinkage on the dose distribution in four cases.

**Case presentation:**

In all cases, we implanted ^125^I sources using a modified peripheral loading pattern for seed placement. The prescribed dose for each implant was 110 Gy. After delivering the sources, a hydrogel spacer was injected. All cases underwent external-beam radiation therapy approximately 1–2 months after brachytherapy. The prescribed dose of external-beam radiation therapy was 45 Gy in 1.8-Gy fractions. Magnetic resonance imaging was performed for evaluation on the day following seed implantation (baseline), at external-beam radiation therapy planning, and during external-beam radiation therapy. The median hydrogel spacer volume was 16.2 (range 10.9–17.7) cc at baseline, 14.4 (range, 9.4–16.1) cc at external-beam radiation therapy planning, and 7.1 (range, 2.0–11.4) cc during external-beam radiation therapy. The hydrogel spacer volume during external-beam radiation therapy was significantly lower than that at external-beam radiation therapy planning. The rectum V60–80 (rectal volume receiving at least 60–80% of the prescribed dose of external-beam radiation therapy) during external-beam radiation therapy was significantly higher than that at external-beam radiation therapy planning.

**Conclusions:**

The potential reduction in hydrogel spacer size during external-beam radiation therapy following brachytherapy can lead to unexpected irradiation to the rectum. This case presentation would be helpful for similar cases.

## Background

Brachytherapy (BT) is a well-established standard treatment option for localized prostate cancer [1–3]. Lately, increasing evidence has suggested a role for BT in high-risk prostate cancer due to technological advances [4, 5]. In high-risk prostate cancer, good outcomes have been reported for the combination of androgen-deprivation therapy (ADT) and external-beam radiation therapy (EBRT) following BT [5]. However, there is concern regarding the relatively high rectal adverse events associated with the combination of EBRT and BT.

Recently, the use of hydrogel as a spacing material (Space OAR system; Augmenix, Waltham, MA) has attracted attention. Hydrogel spacers expand the perirectal space as an injected liquid and polymerize into a soft absorbable spacer to increase the distance between the rectum and prostate, reducing the effects of irradiation to the rectum. Several studies have reported the effectiveness of hydrogel spacers [6–8].

On the other hand, there is concern regarding hydrogel spacer shrinkage during EBRT after BT. Hydrogel spacers reportedly retain their shape for approximately 3 months after insertion [7], but it is unclear whether the spacers retain their shape from the time of insertion until the end of EBRT. Hydrogel spacer shrinkage during EBRT could affect the dose distribution, including unexpected irradiation to the rectum. However, few studies have assessed hydrogel spacer shrinkage during EBRT after BT and hydrogel spacer injection.

Therefore, this case presentation evaluates the changes in hydrogel spacer appearance by magnetic resonance imaging (MRI) during EBRT after BT for prostate cancer and analyzed the effect of this shrinkage on treatment in four cases.

### Case 1

The patient was a 74-year-old Japanese man with localized high-risk prostate cancer (T2bN0M0, Union for International Cancer Control 8th edition). The combined Gleason score (GS) was 9, and the initial prostate-specific antigen (PSA) concentration was 9.4 ng/mL. Patients with high-risk disease received ADT and BT followed by EBRT. Our implant procedure was previously described in detail [9]. We implanted the ^125^I sources using a modified peripheral loading pattern for seed placement. After delivering the sources, a hydrogel spacer was injected into the perirectal fat to displace the anterior rectal wall posteriorly away from the prostate. A post-implantation dosimetric analysis was performed by computed tomography (CT) 30 days after implantation. The target volume of EBRT was defined as the prostate plus the seminal vesicle with adequate margins. The prescribed dose of EBRT was 45 Gy, with a daily fraction of 1.8 Gy. In this case, the time of EBRT start was 47 days after BT, while the time of EBRT termination was 79 days after BT. The time of MRI during EBRT was 76 days after BT (the 22nd EBRT treatment). The hydrogel spacer volume was 15.0 cc at baseline, 14.4 cc at EBRT planning, and 11.4 cc during EBRT. During EBRT, the hydrogel spacer had shrunk slightly. On the other hand, the perirectal distance (from the prostate to the anterior rectum at the middle height of the prostate) did not change much, with a value of 22.1 mm at baseline, 19.9 mm at EBRT planning, and 20.7 mm during EBRT. The rectum V100, V90, V80, V70, and V60 (rectum V*X* being defined as the rectal volume receiving at least *X*% of the prescribed dose of EBRT) at EBRT planning and during EBRT were 0 and 0.2 cc, 1.0 and 1.5 cc, 1.5 and 2.6 cc, 2.0 and 3.5 cc, and 3.2 and 4.6 cc, respectively. In case 1, a slight reduction of the hydrogel spacer was observed, which resulted in a slight increase in the dose to the rectum.

### Case 2

**Fig. 1:**
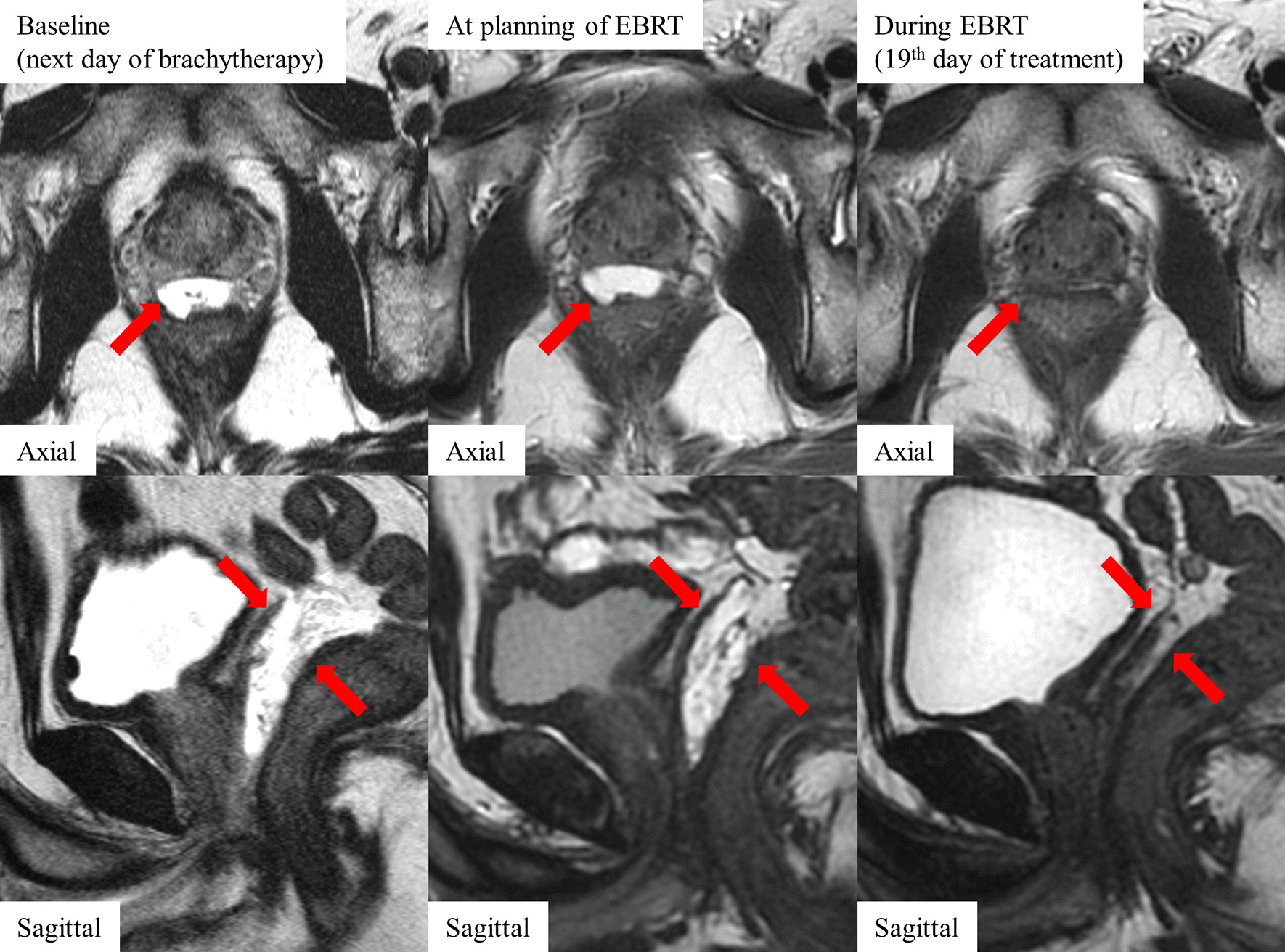
Successive change in the hydrogel spacer in case 2. Red arrows indicate the hydrogel spacer. The hydrogel spacer was successfully injected and had retained its shape during the planning for external-beam radiation therapy (EBRT). On the 19th day of EBRT, the hydrogel spacer had been absorbed and the rectum came into contact with the prostate

The patient was a 56-year-old Japanese man with localized high-risk prostate cancer (T2aN0M0, GS 8, PSA 6.3 ng/mL). He also received ADT and BT followed by EBRT. The time of EBRT start was 68 days after BT, while the time of EBRT termination was 103 days after BT. The time of MRI during EBRT was 93 days after BT (the 19th EBRT treatment). The hydrogel spacer volume was 17.4 cc at baseline, 12.9 cc at EBRT planning, and 2.0 cc during EBRT. The perirectal distance was 11.4 mm at baseline, 6.5 mm at EBRT planning, and 0.6 mm during EBRT. The hydrogel spacer had retained its shape during the planning for EBRT. However, on the 19th day of EBRT, the hydrogel spacer had been shrinking rapidly. Figure 1 shows the successive shrinkage of the hydrogel spacer in case 2. The rectum V100, V90, V80, V70, and V60 at EBRT planning and during EBRT were 0.1 and 3.6 cc, 3.0 and 7.1 cc, 5.5 and 8.8 cc, 7.0 and 10.1 cc, and 8.7 and 11.7 cc, respectively. In case 2, the hydrogel spacer shrinkage during EBRT caused unexpected irradiation to the rectum.

### Case 3

The patient was a 65-year-old Japanese man with localized high-risk prostate cancer (T2aN0M0, GS 8, PSA 8.8 ng/mL). The time of EBRT start was 53 days after BT, while the time of EBRT termination was 88 days after BT. The time of MRI during EBRT was 74 days after BT (the 16th EBRT treatment). The hydrogel spacer volume was 10.9 cc at baseline, 9.4 cc at EBRT planning, and 7.1 cc during EBRT. The perirectal distance was 14.5 mm at baseline, 13.6 mm at EBRT planning, and 11.8 mm during EBRT. The hydrogel spacer shrank gradually during treatment period. The rectum V100, V90, V80, V70, and V60 at EBRT planning and during EBRT were 0 and 0.4 cc, 1.1 and 3.1 cc, 2.5 and 4.6 cc, 4.1 and 5.7 cc, and 6.0 and 7.1 cc, respectively. In case 3, gradual reduction of the hydrogel spacer and a slight increase in the dose to the rectum were observed.

### Case 4

**Fig. 2:**
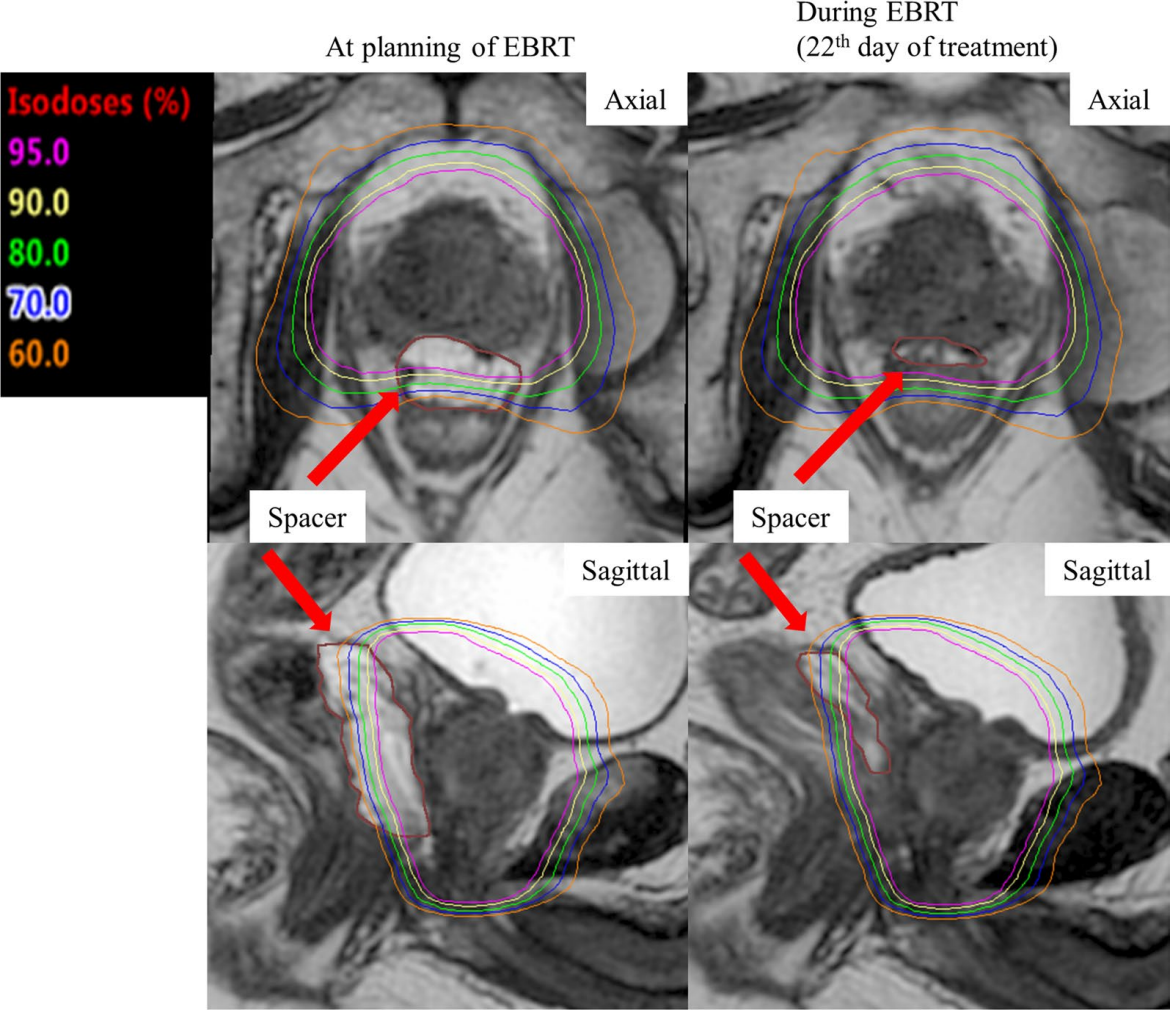
Dose distributions at treatment planning and during external-beam radiation therapy (EBRT) following brachytherapy in case 4. Red arrows indicate the hydrogel spacer (brown line). The rectum was not covered by high doses due to the hydrogel spacer during EBRT planning. The hydrogel spacer shrank during EBRT, causing the rectal anterior wall to be included in the high-dose area

The patient was a 72-year-old Japanese man with localized high-risk prostate cancer (T3aN0M0, GS 8, PSA 14.6 ng/mL). The time of EBRT start was 68 days after BT, while the time of EBRT termination was 104 days after BT. The times of MRI during EBRT were 83 and 99 days after BT (the 10th and 22nd EBRT treatment). The hydrogel spacer volume was 17.7 cc at baseline, 15.8 cc at EBRT planning, 11.4 cc at first evaluation during EBRT, and 4.5 cc at second evaluation during EBRT. The perirectal distance was 15.0 mm at baseline, 15.0 mm at EBRT planning, 10.5 mm at first evaluation during EBRT, and 4.1 mm at second evaluation during EBRT. The rectum V100, V90, V80, V70, and V60 at EBRT planning, first and second evaluation during EBRT were 0, 0 and 0.9 cc, 0, 0 and 2.3 cc, 0.1, 0.4 and 3.2 cc, 0.3, 2.2 and 4.1 cc, and 0.6, 3.6 and 5.0 cc, respectively. In case 4, between the first and second evaluation during EBRT, the hydrogel spacer shrank significantly. Correspondingly, an increase in the dose to the rectum was observed. Figure 2 shows the dose distributions at EBRT planning and during EBRT following BT in case 4.

**Fig 3.**
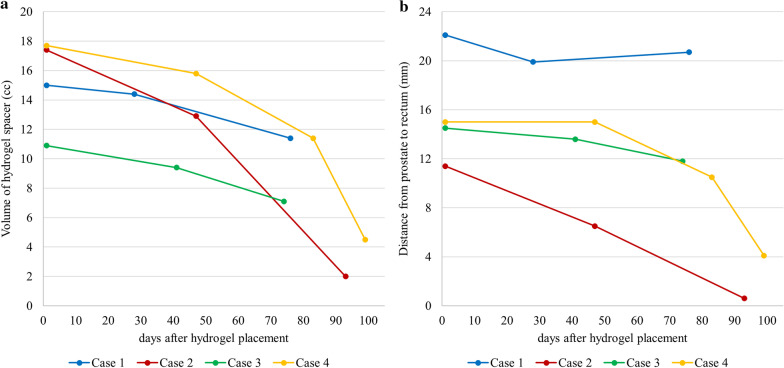
Successive changes in **a** hydrogel spacer volume and **b** distance from prostate to rectum

**Fig 4.**
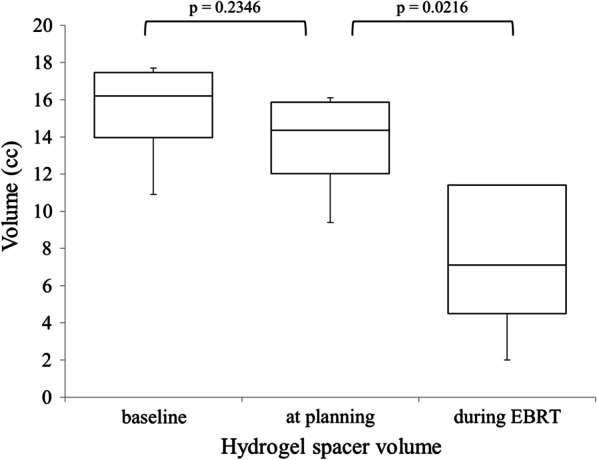
Hydrogel spacer volume changes during external-beam radiation therapy (EBRT). No significant change in volume was observed between baseline (the next day after brachytherapy) and at EBRT planning. The hydrogel spacer volume during EBRT was significantly decreased compared with at treatment planning.

**Fig 5.**
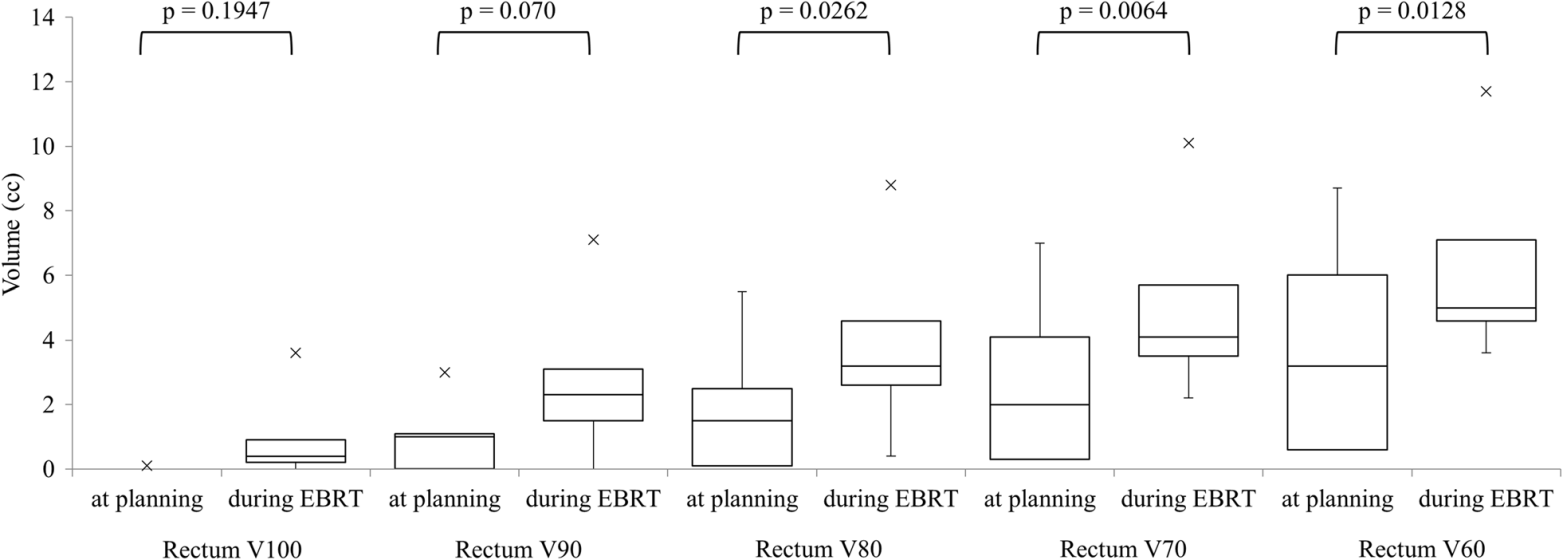
Effect of hydrogel spacer on external-beam radiation therapy (EBRT); comparison of irradiated rectal volume between treatment planning and during EBRT. Rectum V*X* is defined as the rectal volume receiving at least *X*% of the prescribed dose of EBRT. The rectum V60–80 during EBRT were significantly higher than at treatment planning

Patient characteristics are summarized in Table 1. Table 2 presented the details of hydrogel spacer shrinkage during EBRT after BT and its effects on EBRT planning. The median hydrogel spacer volume at baseline was 16.2 (range 10.9–17.7) cc. The median perirectal distance at baseline was 14.8 (range 11.4–22.1) mm. The median hydrogel spacer volume was 14.4 (range 9.4–16.1) cc at EBRT planning and 7.1 (range 2.0–11.4) cc during EBRT (Fig. 3). The hydrogel spacer volume during EBRT was significantly lower than that at EBRT planning, while no significant change in volume was observed between baseline and at EBRT planning (Fig. 4). The median perirectal distance was 14.3 (range 6.5–19.9) mm at EBRT planning and 10.5 (range 0.6–20.7) mm during EBRT (Fig. 3). The median rectum V100, V90, V80, V70, and V60 at EBRT planning and during EBRT were 0 and 0.4 cc, 1.0 and 2.3 cc, 1.5 and 3.2 cc, 2.0 and 4.1 cc, and 3.2 and 5.0 cc, respectively. The rectum V80–60 during EBRT were significantly higher than those at EBRT planning (Fig. 5). However, the dose to the PTV showed little change. In the above analyses, rectum refers to the rectal wall, which was defined based on the rectum contours with 4-mm thickness. The significant differences in hydrogel spacer volume and the effect on the dose distributions between baseline and at EBRT planning and between EBRT planning and during EBRT were evaluated by Student’s *t*-tests. BellCurve for Excel (version 3.20; Social Survey Research Information Co., Ltd., Tokyo, Japan) was used to perform statistical analyses. Statistical significance was defined as *p* < 0.05.

**Table 1:** Patient characteristics

	Age (years)	Prostate volume	ADT	Dose of BT	Number of implanted seeds	Dose of EBRT	EBRT starting date from BT	EBRT termination date from BT	Timing of evaluation from BT (*X*th day of EBRT)
Case 1	74	19.5 cc	Yes	110 Gy	29	45 Gy/25 fractions	47	79	76 (22nd)
Case 2	56	26.4 cc	Yes	110 Gy	37	45 Gy/25 fractions	68	103	93 (19th)
Case 3	65	28.6 cc	Yes	110 Gy	42	45 Gy/25 fractions	53	88	74 (16th)
Case 4	72	25.8 cc	Yes	110 Gy	39	45 Gy/25 fractions	68	104	83 and 99 (10th and 22nd)

ADT, androgen-deprivation therapy; BT, brachytherapy; EBRT, external-beam radiation therapy.

**Table 2:** Details of hydrogel spacer shrinkage and its effects on external-beam radiation therapy planning

		Days after hydrogel injection	Hydrogel spacer		PTV dose (Gy)			Irradiated rectum volume (cc)^a^				
			Distance (mm)	Volume (cc)	Mean	Maximum	Minimum	V100	V90	V80	V70	V60
Case 1	Baseline	1	22.1	15.0								
	Planning of EBRT	28	19.9	14.4	45.2	48.2	32.2	0	1.0	1.5	2.0	3.2
	Evaluation during EBRT	76	20.7	11.4	45.2	48.2	32.2	0.2	1.5	2.6	3.5	4.6
Case 2	Baseline	1	11.4	17.4								
	Planning of EBRT	47	6.5	12.9	45.2	47.9	36.1	0.1	3.0	5.5	7.0	8.7
	Evaluation during EBRT	93	0.6	2.0	45.1	47.9	36.1	3.6	7.1	8.8	10.1	11.7
Case 3	Baseline	1	14.5	10.9								
	Planning of EBRT	41	13.6	9.4	45.1	47.5	37.7	0	1.1	2.5	4.1	6.0
	Evaluation during EBRT	74	11.8	7.1	45.2	47.5	37.7	0.4	3.1	4.6	5.7	7.1
Case 4	Baseline	1	15.0	17.7								
	Planning of EBRT	47	15.0	15.8	45.0	47.5	39.6	0	0	0.1	0.3	0.6
	Evaluation during EBRT	83	10.5	11.4	45.0	47.5	39.6	0	0	0.4	2.2	3.6
	Second evaluation during EBRT	99	4.1	4.5	45.0	47.5	39.6	0.9	2.3	3.2	4.1	5.0

PTV, planning target volume; BT, brachytherapy; EBRT, external-beam radiation therapy.

^a^Rectum V*X* defined as the rectum volume receiving at least *X*% of the prescribed dose of EBRT

## Discussion and conclusions

Hydrogel spacers are excellent injectable agents that have the potential to reduce rectal adverse events [6–8]. We believe that hydrogel spacers are useful in combination with BT and EBRT for high-risk prostate cancer cases as the incidence of gastrointestinal toxicity for the combination of BT and EBRT is higher than that for EBRT alone. The 5-year cumulative incidence of grade 3 gastrointestinal events was 8.1% for the combination of BT and EBRT, versus 3.2% for EBRT alone, while the 9-year biochemical progression-free survival estimates were 83% for BT plus EBRT versus 62% for EBRT alone [5, 10]. Therefore, hydrogel spacers may reduce the risk of rectal adverse events while providing good tumor control. Indeed, several studies have reported the usefulness of hydrogel spacers in patients with prostate cancer undergoing combination BT and EBRT [11–13]. However, it was unclear whether the shape of the hydrogel spacer was maintained during EBRT after BT because it was naturally absorbed over time after insertion. This case report observed a reduction in hydrogel spacer size during EBRT treatment as well as unexpected irradiation to the rectum. This case presentation is unique in evaluating the change in the appearance of hydrogel spacers by MRI during EBRT after BT for high-risk prostate cancer and analyzing the effect of this shrinkage on the dose distribution. Since few studies have reported the influence of hydrogel spacer shrinkage during EBRT on dose distribution, this case presentation is helpful for similar cases.

The hydrogel spacer reduction mainly depended on the duration from insertion. Hydrogel spacers remain in place for 3 months during radiation treatment and are then absorbed and excreted through the patients’ urine. Other studies have reported changes in hydrogel spacers during treatment [7, 14]. Mariodas *et al.* reported a mean perirectal distance in the hydrogel spacer group of 9.0 mm at 3 months following insertion. Hydrogel absorption was evident during the 3-month imaging, with a mean space volume in patients imaged the week before their 3-month date of 10.9 mm, compared with 6.8 mm the week after [7]. Van Gysen *et al.* reported mean anterior–posterior hydrogel diameters of 1.6 and 1.5  cm at the start and end of treatment, respectively [14]. These two studies reported consistent prostate rectal separation throughout treatment. However, all of our patients experienced spacer shrinkage during EBRT and the irradiation to the rectum was increased compared with that of the initial planning (at 2.5 months after spacer insertion in the earliest case). Van Gysen *et al.* also described evidence of changes consistent with gel resorption in post-treatment MRI of two patients [14]. Although our patients started EBRT later than usual (median of 2 months after insertion), hydrogel spacer shrinkage and its effect on treatment should be considered to avoid underestimation of the rectal doses. To avoid the effects on the dose distribution due to hydrogel spacer shrinkage, EBRT should be completed within 2.5 months after hydrogel spacer insertion. If the completion of EBRT might exceed 2.5 months after insertion, re-evaluation of the hydrogel spacer and treatment planning during EBRT is required.

In conclusion, about 2.5 months from insertion of the hydrogel spacer, the potential reduction in the hydrogel spacer size can cause unexpected irradiation to the rectum. Therefore, EBRT should be planned to end within 2.5 months after hydrogel spacer insertion or, in cases in which EBRT completion may exceed 2.5 months, it is important to evaluate hydrogel spacer shrinkage and treatment planning during EBRT. Hydrogel spacers are useful and are expected to be increasingly used worldwide in the future. This case presentation provides important insight regarding treatment accuracy.

## Data Availability

The datasets are available in this report and are available from the corresponding author on reasonable request.

## Abbreviations

BT: Brachytherapy
ADT: Androgen-deprivation therapy
EBRT: External-beam radiation therapy
MRI: Magnetic resonance imaging
CT: Computed tomography
PTV: Planning target volume
CBCT: Cone-beam computed tomography
GS: Gleason score
PSA: Prostate-specific antigen
